# B Cell Aplasia Following CAR T Cell Therapy: Incidence, Kinetics, Prognostic Implications, and Clinical Management

**DOI:** 10.3390/cancers18162680

**Published:** 2026-08-19

**Authors:** Malak Khalifeh, Malini Surapaneni, Huda Salman

**Affiliations:** 1 Brown Center for Immunotherapy, Melvin and Bren Simon Comprehensive Cancer Center, School of Medicine, Indiana University (IU), Indianapolis, IN 46202, USA; mkhalife@iu.edu; 2 Melvin and Bren Simon Comprehensive Cancer Center, Division of Hematology and Oncology, School of Medicine, Indiana University (IU), Indianapolis, IN 46202, USA; msurapan@iu.edu

**Keywords:** B cell aplasia, CAR T cell therapy, CD19, hypogammaglobulinemia, minimal residual disease, relapse, immunoglobulin replacement, tisagenlecleucel, axicabtagene ciloleucel

## Abstract

B cell aplasia (BCA), the sustained depletion of circulating CD19-positive B cells, is the defining on-target, off-tumor consequence of anti-CD19 chimeric antigen receptor (CAR) T cell therapy. Despite its occurrence across all approved CAR T cell products and all responding patients, BCA has not been systematically reviewed as a standalone clinical and biological phenomenon. This review synthesizes data from landmark trials and real-world cohorts across B cell-acute lymphoblastic leukemia (B-ALL), large B cell lymphoma, follicular lymphoma, mantle cell lymphoma, and chronic lymphocytic leukemia to characterize BCA incidence, recovery kinetics, prognostic significance, and management implications. Anti-BCMA products, which mechanism of action differs fundamentally, depleting plasma cells rather than B cell precursors, are intentionally excluded from this review. BCA is observed in all responders and is consistently absent in non-responders across reported cohorts , making it a reliable pharmacodynamic marker of CAR T cell activity. Its prognostic significance is disease-specific: in lymphoma, BCA recovery does not predict relapse and durable remission is achievable independent of sustained aplasia; in B-ALL, early BCA recovery within six months is a robust independent predictor of CD19-positive relapse, while persistent BCA correlates with sustained remission. CD19-negative antigen-escape relapse occurs preferentially in the presence of intact BCA and high pre-infusion tumor burden. Combining BCA kinetics with bone marrow next-generation sequencing minimal residual disease assessment at day 28 and month 3 constitutes the most powerful post-infusion risk-stratification framework currently available. BCA is mechanistically dissociated from hypogammaglobulinemia: IgM declines rapidly and profoundly, IgA more slowly, while IgG—maintained by CD19-negative long-lived plasma cells—is the most preserved isotype. No B cell count threshold below which hypogammaglobulinemia becomes clinically significant has been established. Evidence-informed IVIG replacement thresholds are proposed, though no randomized trial data exist to support them, representing a critical gap requiring prospective investigation.

## Simple Summary

CAR T cell therapy targeting CD19 reliably wipes out patients’ normal B cells, a side effect called B cell aplasia (BCA). This review shows that across leukemia and lymphoma, BCA is actually a useful sign the treatment is working—it appears in all responders and is absent in non-responders. Its meaning for long-term outcomes differs by disease: in lymphoma, whether B cells recover does not predict relapse, but in acute lymphoblastic leukemia, B cells returning too early (within six months) signals higher relapse risk, while prolonged suppression is reassuring. BCA is also mechanistically separate from the drop in antibody levels some patients experience—the three main antibody types are affected differently, with IgG holding up best. Lastly, there is no established B cell threshold for when to start antibody replacement therapy (IVIG); the authors propose practical guidelines but acknowledge real trial evidence is still lacking.

## 1. Introduction

Ten chimeric antigen receptor (CAR) T cell products approved by the US FDA targeting CD19 and B cell maturation antigen (BCMA) have transformed treatment of hematological malignancies, including B cell-acute lymphoblastic leukemia (B-ALL), large B cell lymphoma (LBCL), follicular lymphoma, mantle cell lymphoma (MCL), chronic lymphocytic leukemia (CLL), and multiple myeloma [1,2,3]. The on-target, off-tumor elimination of normal B lymphocytes—a consequence of shared CD19 expression between malignant and healthy B cells—represents an important and incompletely characterized toxicity. B cell aplasia (BCA), defined as sustained depletion of circulating B cells, arises predictably in responding patients and is accompanied by progressive hypogammaglobulinemia with heightened susceptibility to sinopulmonary, encapsulated bacterial, and opportunistic infections [4].

BCA is not unique to CAR T cell therapy: inotuzumab ozogamicin and blinatumomab induce BCA of shorter duration [5]; transient BCA follows systemic chemotherapy [6]; and prolonged BCA has been documented after allogeneic hematopoietic stem cell transplantation (HSCT) [7,8]. What distinguishes post-CAR T BCA is its role as a functional surrogate for CAR T cell persistence and, in B-ALL, as a dynamic predictor of relapse risk. BCA is observed in all responders and is consistently absent in non-responders across reported cohorts [6,7]. This review focuses exclusively on anti-CD19 CAR T cell products. BCMA-directed products (idecabtagene vicleucel, ciltacabtagene autoleucel) are intentionally excluded because their mechanism of immune disruption is fundamentally different: by targeting plasma cells directly, they deplete the primary source of circulating immunoglobulin and produce a pattern of humoral immunodeficiency that is more severe and mechanistically distinct from BCA driven by anti-CD19 therapy. Comparative data on BCA in BCMA-directed products are therefore not synthesized here.

A narrative review of the literature was conducted through the literature search strategy using PubMed/MEDLINE and EMBASE databases from inception through March 2025, using the search terms “B cell aplasia,” “CAR T cell therapy,” “CD19,” “hypogammaglobulinemia,” “immunoglobulin replacement,” “relapse,” and “MRD” in combination. Reference lists of included articles were reviewed for additional relevant publications. Inclusion criteria were: (1) clinical studies reporting BCA incidence, duration, or recovery in patients receiving anti-CD19 CAR T cell therapy; (2) studies reporting immunoglobulin levels or infectious complications in this population; and (3) reviews or consensus statements on management of hypogammaglobulinemia after CAR T cell therapy. Case reports, preclinical studies, and studies focused exclusively on BCMA-directed therapy were excluded. No language restriction was applied. Study selection was performed by two reviewers (M.K., M.S.) with discordances resolved by the senior author (H.S.). Because this is a narrative rather than systematic review, formal PRISMA reporting and meta-analytic pooling were not performed.

BCA is inconsistently reported across trials, with variable definitions and follow-up durations. This review synthesizes BCA incidence, recovery kinetics, and prognostic significance across disease subtypes, with attention to modifying factors and the evidence base for post-infusion management.

## 2. Incidence and Recovery of B Cell Aplasia

BCA develops in all objective responders and is consistently absent in non-responders across disease subtypes and products [9,10]. It should be noted that this relationship, while highly consistent in published series, is based on observational data rather than a biological absolute, and rare exceptions may exist in underpowered or selected cohorts. Recovery kinetics depend on the malignancy, CAR T product, and host immune environment [11]. In B-ALL cohorts, early recovery within six months is observed in a substantial binary in all responders.

A proportion of patients experience slow recovery beyond 6 months, with some patients maintaining aplasia for several years [1,12]. This estimate is derived primarily from B-ALL data and should not be extrapolated to lymphoma subtypes, where recovery rates and timeframes differ substantially (see disease-specific sections below).

### 2.1. Large B Cell Lymphoma

In ZUMA-1, B cell recovery occurred in 75% of assessable patients with ongoing responses at 24 months, while 34% (11/32) maintained ongoing responses despite loss of detectable gene-marked CAR T cells, suggesting that durable remission in large B cell lymphoma does not require prolonged CAR T-cell persistence or sustained B cell aplasia [13]. At three years, peripheral B cells were detectable in all evaluable patients with ongoing responses, and 91% (21/23) demonstrated polyclonal B cell recovery and diversity, with a median Igκλ ratio of 1.6 [14]. The 5-year ZUMA-1 analysis consequently concluded that protracted B cell aplasia was not required for durable responses [2].

These observations were corroborated in the phase 3 ZUMA-7 trial, in which B cell recovery progressively increased after axi-cel therapy: B cell aplasia was present in 62.3% of evaluable patients at three months but in only 22.6% at 24 months. B cell levels began to recover as circulating CAR T cell levels declined to very low or undetectable levels, while durable clinical benefit was maintained [13]. Together, ZUMA-1 and ZUMA-7 demonstrate that durable disease control following axi-cel can persist despite B cell recovery and loss of detectable circulating CAR T cells, indicating that neither indefinite CAR T cell persistence nor sustained B cell aplasia is necessary for long-term remission [2,13,14].

Immunoglobulin recovery lags substantially behind B cell reconstitution. Among tisagenlecleucel-treated patients not receiving IVIG, IgM normalized in 40% by 12–24 months, IgG in 30% at 18 months, and IgA in only 38% at five years [15,16]. In JCAR017 (lisocabtagene maraleucel)-treated patients with relapsed/refractory non-Hodgkin lymphoma, 92% developed BCA after infusion, with 86% and 73% maintaining aplasia at six and 12 months [17].

### 2.2. Follicular Lymphoma

In ZUMA-5 (*n* = 109), all four non-responding patients lacked BCA, while objective responders showed progressive B cell recovery: 41% and 48% at six and 12 months, increasing to 69% at 18 months [10]. In a separate cohort, all patients achieving complete conversion from partial response maintained sustained BCA, with only one demonstrating B cell recovery at one year [18].

### 2.3. Mantle Cell Lymphoma

In the phase 2 KTE-X19 trial in relapsed/refractory MCL (*n* = 60, 67% CR rate), all objective responders developed BCA while non-responders did not; B cell recovery occurred in 62% (21/34) of patients with ongoing response at six months [3]. Seven of eight patients who subsequently relapsed recovered B cells at six months, and two non-responders at relapse were CD19-negative [3,10]. CAR transgene was undetectable in 18% of patients with B cell recovery who remained in remission, confirming that BCA recovery does not preclude durable disease control in MCL [3].

### 2.4. Chronic Lymphocytic Leukemia

Data in CLL are limited to small series. Complete B cell eradication occurred within 10 weeks in all four treated patients; one patient demonstrated polyclonal B cell recovery at 13 months while maintaining remission at 23 months, confirming that non-malignant B cell precursor reconstitution is achievable [19].

### 2.5. B Cell-Acute Lymphoblastic Leukemia

B-ALL is the disease context in which BCA carries the greatest prognostic weight, developing in patients who achieve remission. In ELIANA (*n* = 75), with 81% response rate and universal immunoglobulin replacement, the probability of persistent BCA was 71% at 12 months and 59% at 24 months (median BCA duration 35.3 months; reported as Kaplan–Meier probability of persistent aplasia), with response duration tightly correlated with tisagenlecleucel persistence and sustained BCA [1,20]. In a phase 2 cohort of 43 pediatric B-ALL patients (93% CR), 45% relapsed with a median BCA duration of only three months and relapse at a median of six months [21]. In ZUMA-3 (*n* = 55 adult R/R B-ALL, 56% CR), responders had deeper BCA than non-responders [22]. For anti-CD22 CAR T cell-treated patients, cumulative incidence of BCA loss was 33%, 48%, and 55% at three, six, and 12 months respectively (median time for recovery was 7.7 months) [23,24].

### 2.6. Extramedullary Leukemia

BCA persistence is shorter in extramedullary B-ALL than bone marrow-predominant disease (median BCA loss at 6.5 months) [25]. The Pediatric Real-World CAR Consortium (PRWCC, *n* = 184) reported a 12-month probability of persistent BCA of 38.9% for non-CNS extramedullary disease versus 59.4% for bone marrow-only disease; CNS disease was comparable to bone marrow-only (66.4%) [26].

## 3. B Cell Aplasia Recovery as a Biomarker of Relapse Risk

In lymphoma, BCA recovery does not predict or predate relapse. In B-ALL, early recovery within six months is a robust independent predictor of CD19-positive relapse, though this relationship is substantially modified by pre-infusion tumor burden and MRD status [12]. CD19-negative relapses occur predominantly in the presence of persistent BCA and functional CAR T cells, particularly in patients with high pre-infusion disease burden (≥5% blasts), reflecting antigen-loss escape driven by sustained CAR T immune pressure rather than CAR T exhaustion [4,12,27].

### 3.1. Duration of BCA and CD19-Positive Relapse

Short BCA duration is the primary risk factor for CD19-positive relapse. Finney et al. demonstrated that 75% of patients with BCA recovery within 63 days had CD19-positive relapse versus CD19-negative recurrence in the majority with BCA > 6 months (log-rank *p* = 0.013) [28]. Summers et al. confirmed that 88% of patients with BCA ≤ 63 days had CD19-positive relapse at recurrence [29]. In 51 tisagenlecleucel-treated R/R B-ALL patients, BCA recovery was an independent predictor of CD19-positive relapse (sub-hazard ratio 21.7, *p* = 0.004), while high tumor burden predicted CD19-negative relapse (SHR 8.35, *p* = 0.004). Median time to BCA loss was 3.1 months in MRD < 10^−2^ patients versus 27.9 months in MRD ≥ 10^−2^ patients [24].

### 3.2. Integration of BCA with MRD: A Dual Biomarker Framework

Pulsipher et al. analyzed 1771 samples from 143 B-ALL patients (ENSIGN/ELIANA) using NGS-MRD [12]. B cell recovery within the first year carried a hazard ratio for relapse of 4.5 (95% CI 2.03–9.97; *p* < 0.001); 22 of 25 (88%) CD19-negative relapses occurred with persistent BCA, while 11 of 14 (79%) CD19-positive relapses followed BCA recovery. Bone marrow NGS-MRD positivity at day 28 and month 3 was independently predictive of relapse (HR = 12, 95% CI 2.87–50; *p* < 0.001). Late BCA recovery (>1 year) carried a more favorable prognosis, with five of six such patients maintaining ongoing response [12].

In a retrospective cohort of 141 B-ALL patients, 37.2% of CD19-positive relapses and 68.3% of CD19-negative relapses occurred with ongoing BCA. Median time to CD19-positive relapse was eight months versus five months for CD19-negative relapse. CD19-negative relapse was more common with prior 4-1BB CAR infusion (*p* = 0.04), younger age < 7 years (*p* < 0.001), and absence of KMT2A rearrangement (*p* = 0.01) [27].

## 4. Factors Modifying BCA Duration

### 4.1. CAR T Cell Persistence

CAR construct design significantly influences BCA duration. 4-1BB-costimulated products (tisagenlecleucel) demonstrate longer in vivo persistence than CD28-costimulated constructs (axicabtagene ciloleucel, brexucabtagene autoleucel), correlating with longer functional BCA [11,30]. In a retrospective comparison, patients receiving 4-1BB-based constructs had significantly longer BCA duration than those receiving CD28-based constructs [31]. Data on whether dual-targeted CAR T cell products produce deeper or longer BCA than single-targeted constructs remain limited to small early-phase series; the phase 1 trial of CD19/CD22 bivalent CAR T cells [32] demonstrated B cell aplasia in responding patients but did not report comparative BCA duration data versus single-antigen products [32,33].

BCA duration is widely used as a functional surrogate for CAR T cell persistence [30]. In B-ALL, early B cell recovery correlates with rapid CAR transgene loss [34] and relapses occurring with persistent BCA and detectable CAR T cells are predominantly CD19-positive (68%, 14/22)—consistent with antigen-loss escape under immune pressure [35]. In lymphoma, important dissociations existing in durable responses have been documented despite CAR T cell disappearance at 4–5 months [36], confirming that BCA recovery does not uniformly imply loss of disease control outside B-ALL [13].

### 4.2. Pre-Infusion Antigen Burden

A marrow CD19-positive load below 15% before lymphodepletion was a significant risk factor for early BCA loss (HR 2.99; 95% CI 1.32–6.81; *p* = 0.005), with median BCA duration of 1.7 months in low-burden versus 6.4 months in high-burden patients (*p* = 0.03) [21,28].

### 4.3. Pre-Infusion MRD Status and Lymphodepletion

Pre-infusion MRD below 10^−2^ was independently predictive of BCA recovery (*p* = 0.03), with median BCA loss at 3.1 versus 27.9 months in high-MRD patients [24]. Fludarabine–cyclophosphamide lymphodepletion was associated with longer BCA duration (6.4 vs 2.1 months with non-fludarabine regimens), though this did not reach statistical significance (*p* = 0.15) [21].

### 4.4. Age and Disease Context

BCA duration is longer in pediatric than adult patients (1-year probability of 48% in one comparative analysis) [37], and 87% of B-ALL relapses in a pediatric cohort were CD19-positive, emphasizing the importance of BCA monitoring in this population [37].

## 5. Clinical Implications and Management Strategies

### 5.1. B Cell Aplasia, Hypogammaglobulinemia, and Infectious Risk

BCA and hypogammaglobulinemia are temporally linked but mechanistically distinct. CD19 is expressed through the plasmablast stage but is absent from long-lived plasma cells—the primary source of circulating IgG [38]. CAR T cells therefore deplete all B cell precursors while largely sparing the existing plasma cell pool, producing a characteristic isotype hierarchy: IgM falls earliest and most profoundly, IgA more slowly, and IgG—maintained by CD19-negative long-lived plasma cells—is the most preserved, particularly in adults with larger plasma cell reserves [39].

Deyà-Martínez et al. prospectively quantified these kinetics in 34 pediatric and young adult B-ALL patients [38]. BCA onset occurred at a median of seven days (range 3–8); 71% reached undetectable IgM by median day 71 (range 41–99) and only 13% reached undetectable IgA by median day 185 (range 11–308). IgG remained normal under empiric IVIG (target trough > 8 g/L from day 28), precluding assessment of the natural nadir—a limitation noted explicitly when interpreting the IVIG thresholds proposed in Table 1. IgM depletion duration correlated significantly with CART19 persistence (373.5 vs 60 days; *p* = 0.001). In adults, hypogammaglobulinemia (IgG < 400 mg/dL) affected 35%, 27%, and 46% of patients at days 15–30, 31–60, and 61–90 respectively, cumulating to 67% beyond day 90 [40] and 90.6% had hypogammaglobulinemia post-infusion in a separate cohort, with even mild hypogammaglobulinemia (IgG 400–600 mg/dL) independently associated with increased mortality [31].

No quantitative B cell count threshold below which hypogammaglobulinemia becomes clinically significant has been established. Because BCA is binary in all responders, immunoglobulin decline is determined by plasma cell reservoir rather than B cell count. The only published threshold—50 cells/μL—is a recovery heuristic signaling when IgG production may resume, not a depletion threshold for harm [44].

Infections occur despite adequate IgG replacement. In the Deyà-Martínez cohort, 19% of patients had infections in the first 28 days (predominantly catheter-related and viral, including HHV-6 encephalitis), and 23% beyond day 28—including *Haemophilus influenzae* meningitis in a fully agammaglobulinemic 2.5-year-old—yet all post-day-28 infections occurred with IgG > 8 g/L, implicating T cell dysfunction and mucosal IgA deficiency as co-determinants [38]. In adult DLBCL cohorts, hypogammaglobulinemia was associated with increased viral but not bacterial or fungal infection risk [45]. Despite near-universal hypogammaglobulinemia, seroprotection for vaccine-preventable pathogens was maintained at approximately 67% (IQR 59–73%) in CD19 CAR T cell recipients—reflecting persistence of pathogen-specific antibodies from long-lived plasma cells—versus substantially lower seroprotection in BCMA-targeted recipients whose therapy destroys plasma cells directly (prevalence ratio 0.47) [43].

### 5.2. IVIG Replacement: Evidence-Informed Clinical Recommendations

All thresholds and recommendations in Table 1 represent expert consensus in the absence of randomized controlled trial data and should be interpreted as author-proposed guidance rather than established clinical guidelines. IVIG replacement lacks an RCT evidence base; current recommendations are extrapolated from primary antibody deficiencies and post-HSCT reconstitution. The strongest available evidence comes from Sutherland et al. (*n* = 579), demonstrating that immunoglobulin replacement was independently associated with decreased mortality after CD19-targeted CAR T cell therapy [31].

For **adults**, expert consensus supports initiating IVIG at IgG ≤ 400 mg/dL within 90 days regardless of infection history, and at 400–600 mg/dL with recurrent or serious infections (target trough ≥ 600 mg/dL; ≥700 mg/dL for recurrent sinopulmonary infections) [40,41,42]. For **pediatric patients**, empiric IVIG (0.5 g/kg/month) should begin at day 28–30, targeting age-adjusted normal IgG with a higher target of ≥800 mg/dL for children under 10 years or those with baseline pulmonary pathology [38,44]. Total IgG is an imperfect surrogate: pathogen-specific antibodies may be preserved even when total IgG is low, while IVIG does not restore mucosal IgA or B cell memory. Decisions on initiation, dosing, and discontinuation should integrate IgG level, infection history, B cell recovery status, and age. Importantly, the Deyà-Martínez et al. cohort [38] used universal empiric IVIG, meaning the natural IgG trajectory in the absence of replacement cannot be determined from their data; the thresholds in Table 1 therefore cannot be fully validated against that study’s IgG values. A practical evidence-graded framework is summarized in Table 1.

Monitoring B cell counts during months 1–12 is essential for early relapse identification, though BCA monitoring alone has limited utility in high-burden patients where CD19-negative relapses predominate [4,12,27,46].

**Risk-Stratified Post-CAR T Management (B-ALL)—Author-Proposed Algorithm; Not an Endorsed Guideline**

▸ All patients: monitor B cells monthly in months 1–12; BM NGS-MRD at day 28 and month 3
▸ BCA loss at <3 months: very high risk; consider allo-SCT; rule out CD19-positive relapse
▸ BCA loss at 3–6 months: consider allo-SCT vs maintenance chemotherapy; intensify MRD monitoring
▸ Persistent BCA + high tumor burden: screen for CD19-negative subclones; FCM antigen-escape surveillance
▸ BCA recovery at >6–12 months: individualized MRD-guided approach
▸ Prior CD19-targeted therapy (e.g., blinatumomab): screen for CD19-negative subclones before and after infusion

### 5.3. Consolidative Allogeneic SCT

Early BCA loss (<6 months) is proposed as an indication for consolidative allo-SCT. Molinos et al. reported that five patients with early BCA recovery who underwent allo-SCT remained in remission, versus four of seven without transplant who relapsed [4]. Median response duration was 12.1 months for BCA < 6 months versus not-reached for BCA > 12 months [20]. Numbers are small and subject to selection bias. For patients with high tumor burden, allo-SCT may also be considered with persistent BCA given CD19-negative escape relapse risk [12,27,30].

### 5.4. Multi-Antigen CAR T Cell Strategies

CD19-negative relapses in patients with persistent CAR T cells reflect the selective pressure of single-antigen targeting. Several clinical programs have evaluated bivalent or sequential CAR T cell constructs to overcome this limitation. Spiegel et al. conducted a phase 1 trial of dual CD19/CD22-targeting CAR T cells in 38 adult patients with relapsed/refractory B cell malignancies, demonstrating a complete remission rate of 52% with manageable toxicity; no CD19-negative relapses were observed in complete responders during the follow-up period of the dose-escalation phase [32]. Shalabi et al. reported phase 1 results of a bicistronic CD19/CD22 CAR T cell product in 26 children and young adults with B-ALL, achieving complete remission in 17 of 26 patients (65%) with limited grade ≥ 3 toxicity [33]. These early-phase data support the biological rationale for dual antigen targeting as a strategy to reduce antigen-escape relapse [47,48]. Larger trials with comparative BCA duration data versus single-antigen products are needed.

### 5.5. Maintenance Strategies for Transplant-Ineligible Patients

Gabelli et al. reported that oral maintenance chemotherapy with or without vincristine/dexamethasone pulses in patients with BCA loss < 6 months ineligible for transplant may extend disease-free intervals [49]. Early BCA loss should prompt active clinical reassessment rather than watchful waiting.

## 6. Limitations

This review has several inherent methodological limitations that should be considered when interpreting its conclusions. First, as a narrative rather than systematic review, study selection was based on expert judgment and may be subject to selection bias; studies with negative or null findings regarding BCA may be underrepresented. Formal meta-analytic pooling was not performed, and no quantitative synthesis of effect sizes is presented. Second, BCA definitions are heterogeneous across included trials: some studies report the proportion of patients with aplasia at fixed timepoints, others report Kaplan–Meier probabilities of persistent aplasia, and others report cumulative incidence of BCA loss—metrics that are not directly comparable. This heterogeneity is acknowledged in Table 2 and Section 6, but limits cross-study comparison throughout. Third, the evidence base for IVIG recommendations (Table 1) is entirely observational or expert consensus; no randomized controlled trial has evaluated IVIG thresholds, dosing, or timing in this population. Fourth, the review focuses exclusively on anti-CD19 products; data from anti-BCMA CAR T cell therapy, where plasma cell depletion produces a more severe and distinct pattern of humoral immunodeficiency, are intentionally not synthesized and the results of this review should not be generalized to that setting. Fifth, the literature base is dominated by trials in pediatric and young adult B-ALL and adult LBCL; data in MCL, FL, and CLL are limited to small series and single trials, and generalizability is accordingly constrained.

## 7. Conclusions

B cell aplasia following CAR T cell therapy is a dynamic biomarker with disease-specific prognostic significance. In B-ALL, BCA duration predicts CD19-positive relapse risk and serves as a functional surrogate for CAR T persistence; early recovery demands urgent reassessment. In lymphoma, BCA recovery does not predict relapse and durable responses are achievable independent of sustained aplasia.

The integration of BCA kinetics with bone marrow NGS-MRD provides the most powerful post-infusion risk-stratification framework available, distinguishing patients at risk for CD19-positive relapse (early BCA recovery + emerging MRD) from those at risk for CD19-negative relapse (persistent BCA + high pre-infusion burden + MRD positivity). Intervention thresholds—allo-SCT, maintenance chemotherapy, or intensified surveillance—should be individualized based on this dual-biomarker framework and patient eligibility.

Critical gaps remain: BCA definitions are inconsistent across trials; no randomized trial has established optimal IVIG thresholds; and no B cell count threshold for clinically significant hypogammaglobulinemia has been defined. Prospective harmonized collection of B cell counts, immunoglobulin isotypes, and paired MRD data should be incorporated as co-primary endpoints in future CAR T cell trials, and a dedicated IVIG randomized trial with infection-free survival as the primary endpoint is overdue.

## Figures and Tables

**Table 1:** Evidence-informed IVIG replacement recommendations following anti-CD19 CAR T cell therapy.

Clinical Scenario	IgG Threshold	Recommendation	Evidence Basis	Level of Evidence
Adults: days 0–90	≤400 mg/dL	Start IVIG 400–500 mg/kg every 3–4 weeks; continue until IgG recovery or B cell reconstitution	Expert consensus; Sutherland et al. [31] . IVIG associated with decreased mortality risk	Retrospective cohort + expert consensus; no RCT
Adults: >day 90 with infections	400–600 mg/dL + recurrent/serious infections	Initiate IVIG; target trough > 600 mg/dL; reassess every 3 months	Wat & Barmettler [41]; Kampouri et al. [42].	Expert consensus; no RCT
Adults: specific antibody deficiency	>600 mg/dL + documented antigen-specific deficiency + recurrent infections	Consider IVIG; screen pathogen-specific titers before initiating	Hill et al. [40].; Walti et al. [43].	Expert consensus; no RCT
Pediatric (all ages)	Below age-adjusted normal; empiric from day 28–30	IVIG 0.5 g/kg/month; target age-adjusted normal; ≥800 mg/dL if age < 10 yr or pulmonary comorbidity	Deyà-Martínez et al. [38].; Doan & Pulsipher [44]	Single prospective cohort + expert consensus; no RCT
All patients: discontinuation	IgG > 600 mg/dL on 2 consecutive measurements + B cells > 50 cells/μL rising serially	Taper IVIG; monitor IgG monthly for 6 months after cessation	Doan & Pulsipher [44]; expert consensus	Expert consensus; no RCT

**Table 2:** Summary of BCA incidence and recovery across CAR T cell trials by disease indication.

Malignancy/Trial	CAR T Product	BCA at 6 mo (%)	BCA at 12 mo (%)	BCA Recovery (%)	Key Finding	BCA Definition Used
LBCL/ZUMA-1 (Ph 1–2)	Axi-cel	77	47	75% at 24 mo	BCA recovery not required for durable response [13]	% with B cell recovery (flow cytometry, threshold NR)
LBCL/ZUMA-1 (Ph 3)	Axi-cel	47	36	77% at 24 mo	Majority recover within two years [14,50]	% with aplasia (CD19+ B cells below institutional LLN)
LBCL (5-yr f/u)	CTL019	—	—	91% at 3 yr	Naïve/memory B cell subsets normalize [2]	% with B cell recovery (any detectable CD19+ B cells)
LBCL/FL/Ph 2a	CTL019	—	—	50% (CR pts)	IgG recovery slower than IgM [16]	% with detectable B cells by flow
FL/ZUMA-5	Axi-cel	59	52	69% at 18 mo	No BCA in non-responders [10]	% still aplastic (B cells < LLN)
MCL/KTE-X19 Ph 2	KTE-X19	38	—	62% (ongoing resp)	BCA loss precedes CD19+ relapse [3]	% with any detectable B cells at timepoint
CLL	Anti-CD19	—	—	1 pt: polyclonal recovery at 13 mo	Continued remission at 23 mo [19]	Complete B cell eradication (qualitative)
B-ALL/ELIANA	Tisagenlecleucel	71% *	59% *	Median 35.3 mo BCA duration	* Probability of persistent BCA [20]	Probability of persistent aplasia by KM estimate (B cells < LLN by flow)
B-ALL/ZUMA-3	KTE-X19	—	—	—	Deeper BCA in CR vs non-CR patients [22]	Depth of aplasia by flow (not threshold-defined)
B-ALL (PRWCC)	Tisagenlecleucel	—	—	38.9% (EML) vs 59.4% (BM-only) at 12 mo	CNS disease not different from BM-only [26]	12-month probability of persistent BCA by KM estimate

## Data Availability

No new data were created or analyzed in this study. Data sharing is not applicable to this article.
